# Sequential bilateral cochlear implant: long-term speech perception results in children first implanted at an early age

**DOI:** 10.1007/s00405-022-07568-4

**Published:** 2022-08-03

**Authors:** F. Forli, L. Bruschini, B. Franciosi, S. Berrettini, F. Lazzerini

**Affiliations:** 1 Otolaryngology, Audiology and Phoniatrics Unit, University of Pisa, Via Paradisa 2, 56100 Pisa, Italy; 2 Department of Clinical Science, Intervention and Technology, Karolinska Institutet, Stockholm, Sweden

**Keywords:** Cochlear implant, Bilateral sequential cochlear implant, Bilateral cochlear implant, Speech perception, Severe-to-profound hearing loss, Congenital hearing loss

## Abstract

**Purpose:**

The study aims to assess the benefit of sequential bilateral cochlear implantation in children with congenital bilateral profound hearing loss, submitted to the first implant at an early age.

**Methods:**

We enrolled all the bilateral sequential cochlear implanted children who received the first implant within 48 months and the second within 12 years of age at our Institution. The children were submitted to disyllabic word recognition tests and Speech Reception Threshold (SRT) assessment using the OLSA matrix sentence test with the first implanted device (CI1), with the second implanted device (CI2), and with both devices (CIbil). Furthermore, we measured the datalogging of both devices. Then we calculated the binaural SRT gain (b-SRTgain) and checked the correlations between speech perception results and the b-SRTgain with the child’s age at CI1 and CI2, DELTA and the datalogging reports.

**Results:**

With the bilateral electric stimulation, we found a significant improvement in disyllabic word recognition scores and in SRT. Moreover, the datalogging showed no significant differences in the time of use of CI1 and CI2. We found significant negative correlations between speech perception abilities with CI2 and age at CI2 and DELTA, and between the SRT with CI1 and the b-SRTgain.

**Conclusions:**

From this study we can conclude that in a sequential CI procedure, even if a short inter-implant delay and lower ages at the second surgery can lead to better speech perception with CI2, children can benefit from bilateral stimulation independently of age at the second surgery and the DELTA.

## Introduction

Simultaneous bilateral cochlear implantation (CI) is the gold standard in the treatment of severe-to-profound bilateral sensorineural hearing loss (SNHL) in children. Compared with unilaterally implanted individuals, bilateral recipients generally show better speech comprehension in noisy conditions, better directional hearing, and an overall improvement in the binaural mechanisms, with an enhancement of the quality of life [1–5]. Furthermore, in the pediatric population, bilateral implantation has been proven to offer gains with respect to speech and language acquisition and learning abilities [6].

A considerable number of children who received a CI in the first years of life are potential candidates for sequential bilateral CI. A global consensus has not yet been established for the indication of a sequential bilateral CI, and many aspects remain controversial. The effective role of prognostic factors predicting the results from the second implant and the benefit from bilateral sequential stimulation has yet to be elucidated. The main variables predicting results of sequential CI in children analyzed in the literature as prognostic factors are the age at the first CI, age at the second CI, the interval between surgeries, and the results with the first implant [1, 7, 8].

This study aims to assess the benefit after a sequential bilateral cochlear implant in children with congenital bilateral profound hearing loss, submitted to the first implant at an early age. The benefit will be measured reporting disyllabic word recognition performances in quiet and in noise and OLSA-test assessed speech reception threshold (SRT). Moreover, in this study we analysed the effect of the main prognostic factors, commonly considered to influence the results from a second sequential CI.

## Materials and methods

We retrospectively reviewed the demographic and audiological data of children submitted to bilateral sequential cochlear implantation at our institution.

We enrolled patients affected by bilateral congenital profound sensorineural hearing loss who received the first implant within 48 months of age and the second within 12 years of age. The follow-up after the second surgery was at least 24 months in all the patients. Patients with cochlear or acoustic nerve malformations without a complete array insertion and with neurodevelopmental disorders were excluded from the study.

Before the first implant, all the children underwent a complete audiological evaluation (including auditory brainstem potentials, otoacoustic emissions, behavioural audiometry with and without hearing aids, tympanometry and stapedial reflex study, speech perception tests with and without hearing aids when possible), and a neuroradiological study of the inner ear, acoustic nerves, and the brain, with a high-resolution computed tomography study and magnetic resonance imaging.

The etiology of hearing loss was investigated by means of blood tests and molecular tests to detect connexine 26 and 30 mutations. When found, the children were submitted to a molecular analysis of the PDS gene or next generation sequencing (NGS) testing.

All the children also underwent an eye examination and a neuropsychiatric evaluation, according to the protocol adopted at our institution [9].

At every audiologic follow-up evaluation, patients underwent a free-field audiometry with the implant. Furthermore, a speech and language therapist assessed the disyllabic word recognition score in silence and with background noise (SNR+10). Finally, in cooperative patients, we evaluated the SRT using the OLSA test [10]. The test was conducted in S0N0 configuration with the noise level fixed at 65dB. If the patients were under 6 years at the evaluation, we used the simplified version of the test [11].

All the evaluation tests were conducted with the first implanted device (CI1) alone, with the second implanted device (CI2) alone, and with both devices (CIbil).

The daily use of each CI was obtained from the manufacturer’s datalogging report.

The time difference between CI1 and CI2 was calculated according to the number of months between the surgeries; we will refer to this data as DELTA.

We compared the speech perception abilities with CI1, CI2 and CIbil. Then we calculated the binaural SRT gain (b-SRTgain) as SRT with CIbil—SRT with CI1.

Furthermore, we tried to correlate the speech perception results and the binaural SRT gain with age at CI1 and CI2, DELTA and the datalogging reports.

### Statistical analysis

The statistical analysis was performed using SPSS 23 software (SPSS, Chicago, IL).

Comparisons for quantitative variables were analyzed with the related samples using the Wilcoxon Signed Rank Test. Univariate correlation for quantitative variables was tested with Pearson correlation. *p* values < 0.05 were considered statistically significant.

## Results

Thirty-six children (14 males, 22 females) were submitted to a bilateral sequential CI at our institution. Among these, we selected 22 patients (7 males, 15 females), according to the previously reported inclusion criteria.

At the study setup, the mean age of the sample was 135.7 months (from 82 to 253 months). The mean age at the first implant (CI1) was 19.3 months (from 10 to 48 months). Nineteen children received CI1 on the right and three children on the left.

The mean age at the second implant (CI2) was 80.4 months (from 28 to 140 months). Three children received CI2 on the right and 19 children on the left.

In all the implanted children, the CI was manufactured by Cochlear^®^. In Table 1 we reported the CI internal parts models used for CI1 and CI2.

**Table 1:** Cochlear implant (CI) internal parts used in our study population

CI1	CI2
1 × CI24R	4 × CI24RE
12 × CI24RE	18 × CI512
9 × CI512	

*CI1* first implanted ear; *CI2* second implant ear

The mean DELTA was 57.3 months (from 14 to 118 months). The mean follow-up after CI1 was 120.6 months (from 78 to 219 months). The mean follow-up after CI2 was 64.3 months (from 24 to 158 months).

The etiology of hearing loss was due to connexin mutations in 12 cases, idiopathic in seven cases, CMV in two cases and meningitis in one case.

The mean disyllabic word recognition score in quiet was 95.5% (from 70% to 100%) with CI1, 73.1% (from 0 to 100%) with CI2 and 98.3% (from 90 to 100%) with CIbil.

The mean disyllabic word recognition score with background noise was 84.8% (from 50% to 100%) with CI1, 59.3% (from 0% to 100%) with CI2 and 90.7% (from 60% to 100%) with CIbil.

The difference of the mean disyllabic word recognition score between CI1 and CI2 is statistically significant both in silence (*p* = 0.002) and with background noise (*p* = 0.002).

The difference of the mean disyllabic word recognition score between CIbil and CI1 is not statistically significant in silence (*p* = 0.067) but it is significant with background noise (*p* = 0.05).

The mean SRT was 3.9 (from −0.5 to 14.4) with CI1, 16.7 (from 0.2 to 60) with CI2 and 2.0 (from −2.1 to 9.6) with CIbil. The mean b-SRTgain was -1.9 (from −8.20 to 0).

**Fig. 1:**
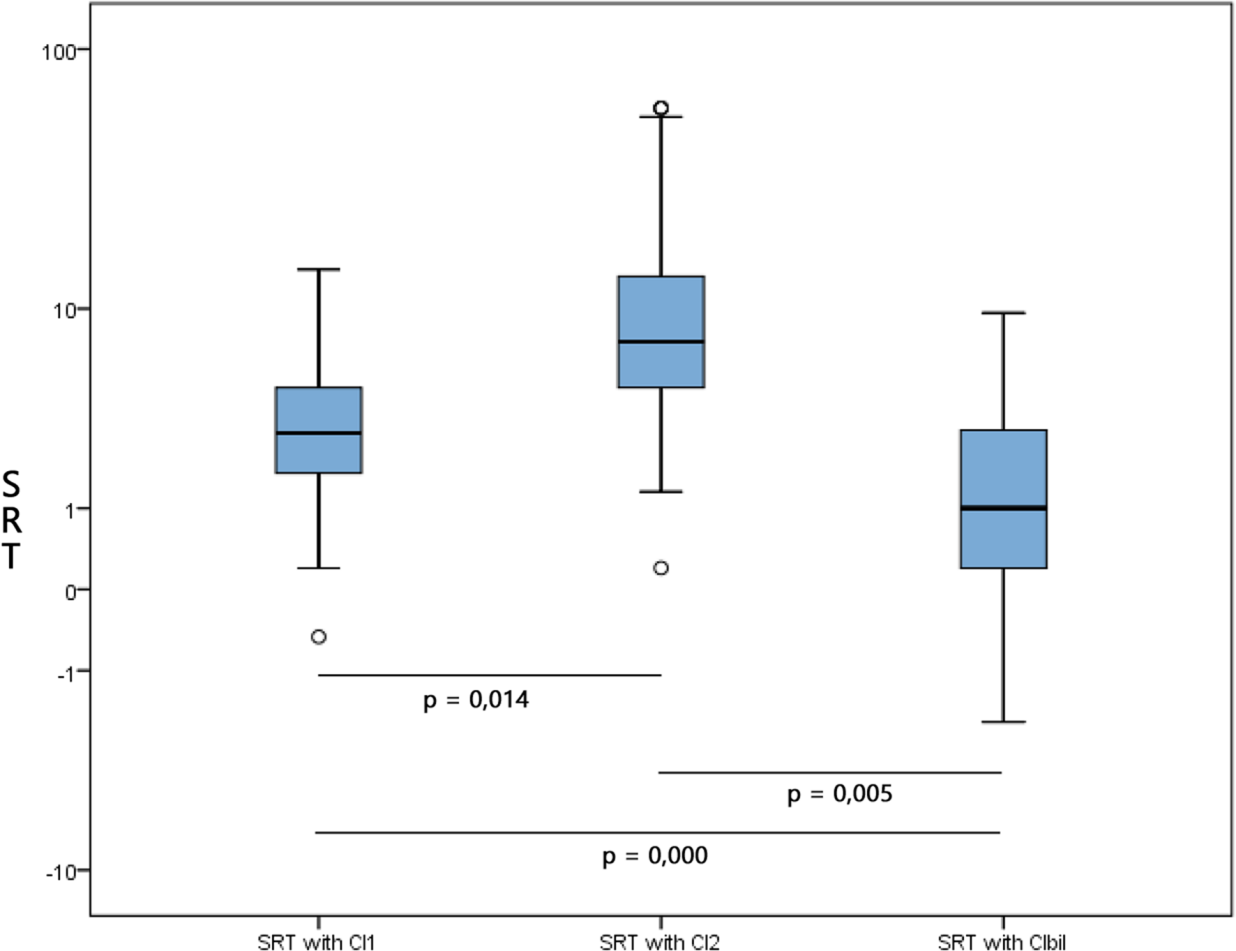
Boxplot showing the mean SRT assessed with CI1, CI2 and CIbil. The difference between SRT with CI1 and CI2 is statistically significant (*p* = 0.014), such as the difference of SRT between CI1 and CIbil (*p* = 0.000) and between CI2 and CIbil (*p* = 0.005)

The difference of SRT between CI1 and CI2 is statistically significant (*p* = 0.014), such as the difference of SRT between CI1 and CIbil (*p* = 0.000) and between CI2 and CIbil (*p* = 0.005) (Fig. 1).

Analysing the datalogging report, the mean daily time of use of CI1 was 12, 81 h (from 7.02 to 14.02 h) and the mean daily time of use of CI2 was 11.66 h (from 5.27 to 14.22). The difference in the time of use between CI1 and CI2 is not statistically significant.

We found a statistically significant negative correlation between the disyllabic word recognition score in silence with CI2 and with age at the second CI (Pearson coefficient = −0.429, *p* = 0.05) and DELTA (Pearson coefficient = −0.561, *p* = 0.008).

**Fig. 2:**
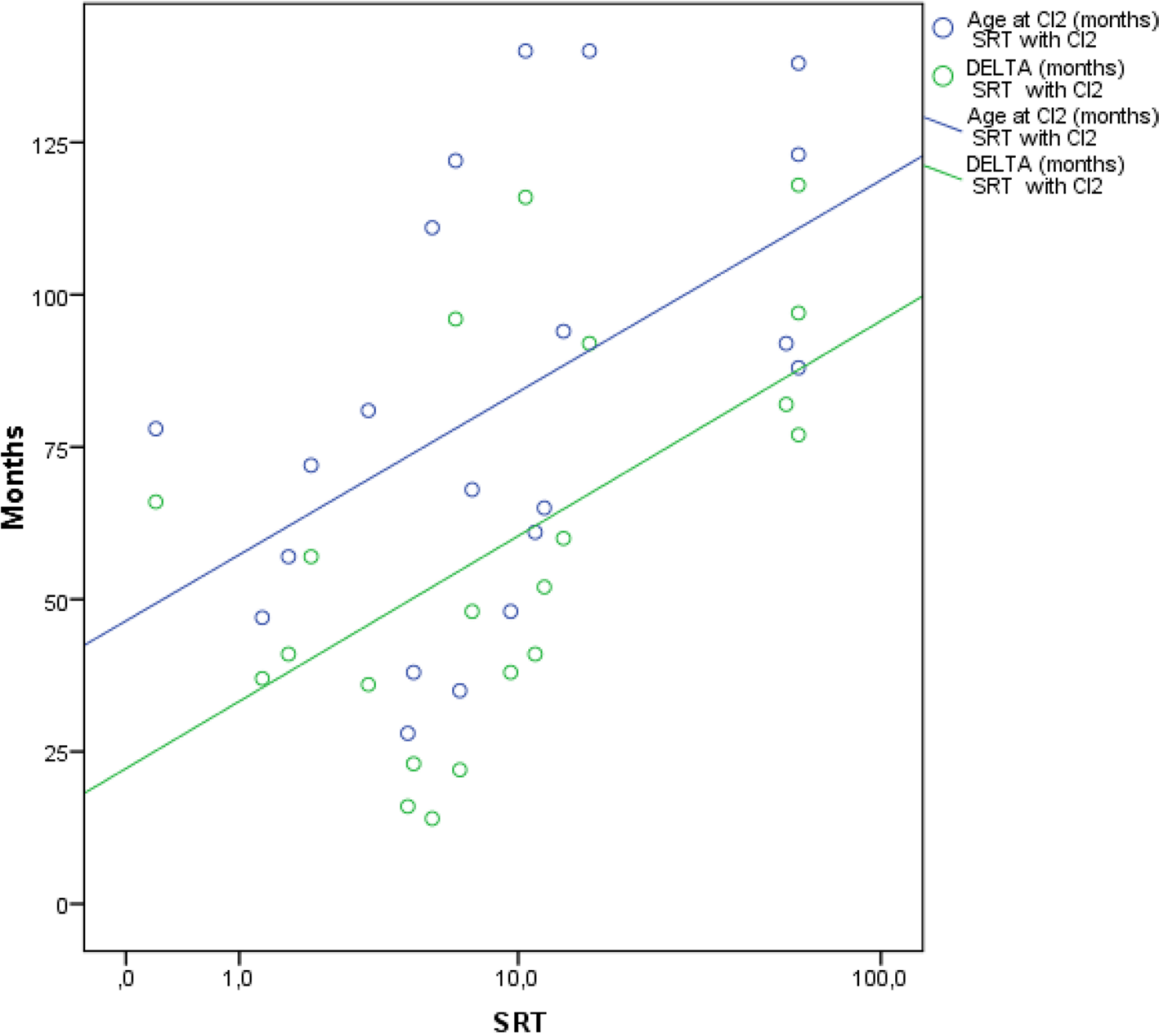
Scatterdot plot showing a statistically significant positive correlation between SRT with C2 and age at CI2 (blue lines and dots) and with DELTA (green lines and dots)

Furthermore, we found a statistically significant positive correlation between SRT with C2 and age at CI2 (Pearson coefficient = 0.463, *p* = 0.035) and with DELTA (Pearson coefficient = 0.599, *p* = 0.004) (Fig. 2).

A statistically significant negative correlation, further, has been found between the SRT with CI1 and the b-SRTgain (Pearson coefficient = −0.665, *p* = 0.001).

We found no correlation between the disyllabic word recognition score in silence and with background noise and SRT with CI1 or with CIbil and age at CI1, the DELTA or the use of hearing aids in the second implanted ear before the operation.

**Fig. 3:**
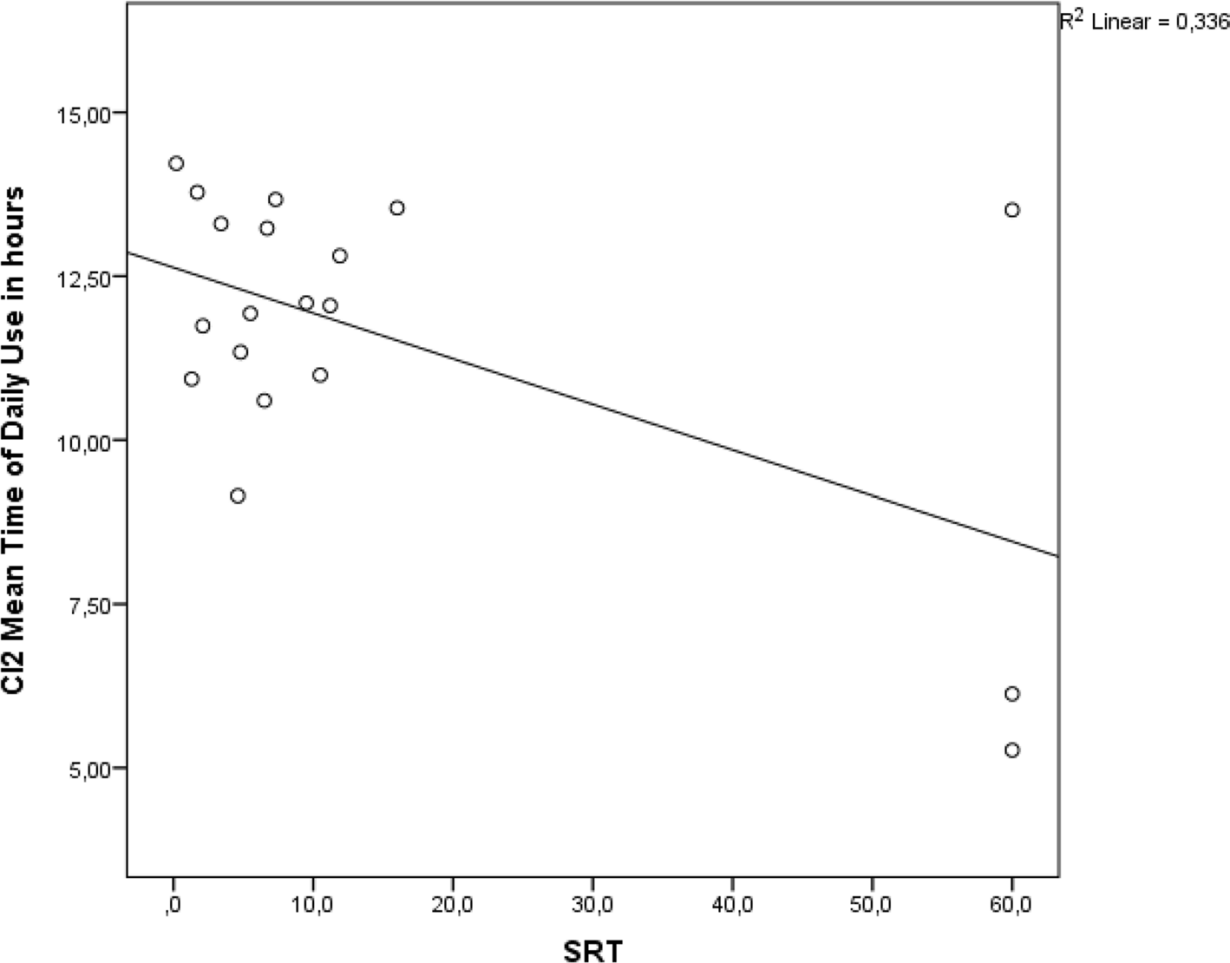
Scatterdot plot showing a statistically significant negative correlation between time of use of CI2 and SRT with CI2

The mean daily time of use of CI2 is significantly positively correlated with the disyllabic world recognition score with CI2 in silence (Pearson coefficient = 0.650, *p* = 0.002), with background noise (Pearson coefficient = 0.661, *p* = 0.001) and negatively with SRT with CI2 (Pearson coefficient = −0.579, *p* = 0.009) (Fig. 3).

On the other hand, no correlation was found between the mean daily time of use of CI2 and the disyllabic world recognition score in silence and with background noise with CIbil, SRT with CIbil, and even b-SRTgain. Nor was a significant correlation found between the mean daily time of use of CI1 and speech perception or SRT results, or the demographic data of the study population.

## Discussion

Simultaneous bilateral cochlear implantation is currently universally accepted as the gold standard for the treatment of congenital bilateral profound SNHL in children and, based on a large amount of evidence in scientific literature, recent international guidelines indicate that it is clinically effective and cost-effective [6, 12]. On the other hand, the possibility to offer a second implant to children previously implanted in one ear, is still controversial and some questions remain open. The recent guidelines by the National Institute for Health and Care Excellence (NICE) (2019), approve bilateral simultaneous CI but not the sequential procedure for children with bilateral severe-to-profound deafness, as this procedure is rated as not cost-effective for the British health system. However, the NICE guidelines admit the possibility of a second implant for previously unilaterally implanted children in situations, where the clinician in charge considers that an additional contralateral CI would provide adjunctive benefit [6]. On the other hand, the guidelines of the French Society of Otolaryngology consider the sequential procedure as an option, recommending a limit to the interval between surgeries [12].

The main issues concern the expected results in the second ear and the adjunctive benefits from bilateral electric stimulation in comparison to the first implant only condition, and mainly it has to be clarified if and eventually to what extent some variables of the child limit the results. Clinical studies in this field have been published, but they are not standardized and are based on inhomogeneous samples, in some cases composed both by adults and children with both prelingual and postlingual onset of hearing loss, with variable degrees of hearing loss and variable ages at implantation [1, 5, 8, 13–17]. Furthermore, the reported results are not reliable, also due to the difficulties in measuring binaural gain, especially in young children. Standard speech perception tests in quiet and in noise are often affected by the ceiling effect, as many children with a unilateral CI perform well both in quiet and in noise. Adaptive tests, as well as tests for directional hearing, may not be applicable to young children.

However, the Authors globally report benefits in speech perception after a sequential bilateral cochlear implantation in children with bilateral profound SNHL, even if lower than for the simultaneous procedure [18, 19]. Moreover, gains in sound localization and in quality of life have been documented [19].

In the present paper we report on a group of children with congenital bilateral profound sensorineural hearing loss, who received the first implant within the fourth year of age and the second implant later in a sequential procedure. In accordance with the literature data, in our study group we recorded positive results after the sequential procedure: all the children regularly use the second implant, and there are no significant differences in the mean daily use between CI1 and CI2 at datalogging analysis.

Furthermore, children achieved significantly higher speech perception scores both in quiet and with background noise while using CIbil than in CI1 condition, and a significantly lower SRT.

Some authors have reported poorer performances when using the CI2 alone, rather than when using CI1 alone. In 2017, Illg reported worse results in speech perception in the second ear than in the first, in a sample of 250 sequentially implanted children [7]; other studies have reported similar data [8, 20].

In our sample, the speech perception results and SRT with CI1 alone were also significantly better than with CI2 alone. None of the children in our study population performed better with CI2 than with CI1 and every child reported to be more confident using the first implant.

Both in the literature and in our sample, despite a substantial benefit from sequential bilateral cochlear implantation, it is noticeable that there is a wide variability of results with CI2; it is reasonable to think that this variability relies on some variable features of the children. To this regard, the main prognostic factors addressed in the literature are the child’s age at CI1, age at CI2, the inter-implant delay (DELTA), the results with CI1 and the use of a hearing aid in the not-implanted ear during the time between surgeries [7, 14, 15, 21–27].

Most of the Authors agree on the importance of an early first implant for the benefit of a second device implanted sequentially, due to the importance of an early cortical stimulation in the maximum neural plasticity period. This is considered to be the main prognostic factor for benefits after CI2 [7, 13]. Most Authors report that the earlier the first surgery, the greater the advantages from the second implant will be, with an optimal period for the first implant within the fourth year of life, in relation to the period of maximum cerebral plasticity [7]. Our group is homogeneous for the precocity of the first implant; every child in the sample received the first implant at a relatively early age (before 4 years of age), and this is probably the reason why we did not find a correlation between age at CI1 and speech perception results with CI2 or CIbil.

In 2019 Jang et al. found that children who were good performers with CI1 achieved functional binaural benefits after the second implantation, irrespective of the inter-implant interval, concluding that sequential CI should be strongly recommended for patients with successful unilateral CI [13].

In our study population, instead, we found a significant negative correlation between the SRT with CI1 and the SRT gain after CI2. Our results seem to be in contrast with the findings proposed by Jang et al., indicating that especially subjects with lower CI1 performances can have a broader benefit by a sequential CI, independently of other factors, such as age at CI2 or DELTA, or the absolute performances with CI2, at least in our population of early first implanted children.

Furthermore, age at the second surgery is considered an important factor, since it conditions the results of the sequential procedure [21]. Most Authors agree that younger age at the second implant leads to better results [1, 7, 8, 15, 21, 26]. Park et al. in 2017, concluded that the results with the second implant are similar to those with the first, if the second implant is done before three and half years of age; however, the Author stated that the sensitive period to achieve good results from the second implant is longer than that for the first implanted ear, up to 12–13 years [21].

We found a statistically significant positive correlation between age at CI2 and SRT with CI2, indicating that the earlier the second implant is done, the better the results will be. On the other hand, we did not find any significant correlation between age at CI2 and the SRT with CIbil. Therefore, we can presume that even if age at CI2 seems to condition the results with CI2 alone, confirming the previous literature findings, this parameter does not seem to have a significant influence on the global results after sequential implantation or on the benefit of using two implants instead of one. However, we must consider that in our study population the mean age at CI2 was only 80.4 months (6.6 years) and none of the subjects had the second implant after 120 months of age (11.6 years).

Another relevant parameter to consider in this area is the length of the inter-implant delay. It is now widely accepted that the best rehabilitative results for bilaterally hearing-impaired children are related to an early bilateral auditory input. In bilaterally deaf children, a unilateral CI would lead to the hearing deprivation of one ear and the onset of the aural preference phenomenon, due to a reorganization of the central auditory areas, which occurs in 2–3 years [27]. Therefore, in children with bilateral profound SNHL unilaterally implanted, it would be important to effectively stimulate the second ear as soon as possible [27]; the lack of an early implantation of the second ear would preclude the possibility of a central integration of the bilateral input, thus compromising the development of binaural abilities [27]. To this regard, the inter-implant delay seems to be a major factor to consider while evaluating the possibility of a second sequential implant [12]. In general, the literature data show that better results are related to short delays between surgeries [14, 15, 23, 24], even if a maximum period beyond which a second implant is not indicated has not been established yet, and some Authors have shown good results from the second implant also after long delays [21, 23]. Recently, Baron et al. reported progress in terms of speech perception in children and adolescents receiving a second cochlear implant after a short interval, but in some cases also after long intervals, concluding that even if it is advisable to keep the interval between surgeries short to enhance the benefits of the second implant, also intervals of several years are not predictive of lack of benefit [1]. Similar considerations are reported by Bianchin et al. [25]. Interestingly, Illg et al. correlated age at first implant and the inter-implant delay to the results, in sequentially implanted children and showed a preferred interval of up to 4 years in children under the age of 4 at first implantation [7]. Benefits in terms of sound localization are also reportedly worse in sequentially implanted congenitally deaf children with long inter-implant delays, as shown by Killan [26].

In our study population, however, we found that DELTA was significantly positively correlated with SRT with CI2, but not correlated with speech perception abilities with CIbil, confirming that even if the inter-implant delay can negatively influence the results with CI2 alone, it does not preclude an overall benefit from bilateral electric stimulation, in terms of speech perception.

We can conclude that in the case of a sequential procedure, even if a short inter-implant delay and lower ages at the second surgery are to be preferred, children can gain benefit from bilateral stimulation also if implanted later.

The use of a hearing aid in the not-implanted ear during the interval between surgeries, could mitigate the hearing deprivation, even if a wide asymmetry between acoustic hearing and electric hearing could preclude the binaurality and lead to a cortical reorganization. Evidence from the literature is limited, but some Authors have found that using a hearing aid between surgeries may enhance the outcomes with the second implant [1, 7, 15–17]. With regard to this, Myhrum et al. reported that a long inter-CI interval was predictive of poor speech perception results with the second CI, but only in the case of children not equipped with hearing aids during the interval [15]. Similar results have been found by Wenrich et al. and by Illg et al. [7, 17].

We did not find a correlation between the use of a hearing aid in the time between the surgeries and the benefits from a second implant. This could be related to the paucity of the sample, the variability in age at the second implant and to the fact that only four children discontinued to use a hearing aid between the surgeries.

The present study presents two main limitations: the first is due to the relative paucity of the sample. The second is related to the complex evaluation of binaural benefit in a pediatric population, where squelching or localization tasks may not be reliably completed.

On the other hand, we have reported the data of a very homogeneous sample, in which every child had a congenital bilateral profound sensorineural hearing loss, received the first implantation at an early age, and had a long follow-up after the second implant.

## Conclusions

According to the literature data, our experience attests that, in children with congenital bilateral profound SNHL, sequential bilateral CI is a clinically effective procedure, allowing them to gain benefits while hearing in quiet and mainly with background noise. Among the possible prognostic factors, in our sample of children with bilateral congenital sensorineural hearing loss who received the first implant early, the inter-implant delay and age at CI2 seem to affect the speech perception outcome using CI2 alone, but do not appear to be correlated with the global benefit of an electrical bilateral stimulation. In other words, we did not find an upper limit of age and of inter-implant delay, that indicated not performing the second implant. These are important factors to keep in mind when evaluating the possibility of a sequential implantation. Sequential bilateral cochlear implantation should, therefore, be strongly considered for unilaterally implanted children who have poor residual hearing or have poor discrimination skills in the contralateral ear. The decision has to be taken on a case-by-case basis, considering that even if in the case of a sequential procedure, a short inter-implant delay and lower age at the second surgery should be preferred, but children can gain benefits from a second implant also if performed later. Older age at the second implant or long inter-implant delays do not preclude obtaining benefits from bilateral electric stimulation.

Further clinical studies, with large and homogeneous samples could contribute to the present knowledge to better define the efficacy of this procedure and the predictive role of the prognostic factors and, consequently, could support decisions not only regarding the clinical aspects, but also economic aspects and cost-efficacy. There is also the necessity to develop more sensitive protocols to assess the binaural benefit after bilateral sequential implantation in children.

## Data Availability

All the data are stored at our clinic’s archives.
